# Crohn’s disease and ulcerative colitis patient-reported outcomes signs and symptoms for the remote management of inflammatory bowel disease during the COVID-19 pandemic

**DOI:** 10.1186/s41687-021-00323-z

**Published:** 2021-06-24

**Authors:** Sergio Pinto, Erica Loddo, Salvatore Paba, Agnese Favale, Fabio Chicco, Sara Onali, Paolo Usai, Massimo Claudio Fantini

**Affiliations:** 1 Department of Medical Science and Public Health, Cagliari, University Hospital of Cagliari, University of Cagliari, Cagliari, Italy; 2 Department of Medical Science and Public Health, Gastroenterology Unit, University of Cagliari, Cagliari, Italy

**Keywords:** Patient-reported outcome, Inflammatory bowel disease, COVID-19, Telemedicine

## Abstract

**Background and aims:**

The COVID-19 pandemic has led to a deep reorganization of hospital services including inflammatory bowel disease (IBD) units. In this situation, conversion of in-person routine follow-up visits into phone consultations might be necessary. Here we explored the feasibility of using the validated Crohn’s Disease (CD) or Ulcerative Colitis (UC) Patient-Reported Outcomes Signs and Symptoms (CD- and UC-PRO/SS) to collect data about abdominal symptoms (abdominal/S) and bowel signs and symptoms (bowel/SS) remotely.

**Methods:**

CD- and UC-PRO/SS were collected during phone consultations and compared among patients with active and inactive disease. The effectiveness of therapeutic intervention in patients with active disease was assessed by PRO/SS variation.

**Results:**

Twenty-one CD and 56 UC patients were evaluated by phone. Six (28.6%) CD and 15 (26.8%) UC patients were considered to have active disease. In CD the bowel/SS but not the abdominal/S module was significantly higher in active patients (mean bowel/SS 2.50 [SE ± 0.44] active vs 0.76 [SE ± 0.18] remission, *p* = 0.008, AUC 0.87; mean abdominal/S 1.11 [SE ± 0.38] active vs 0.24 [SE ± 0.13] remission, *p* = 0.066). UC-PRO/SS measures were significantly higher in active patients as compared to patients in remission (median bowel/SS 1.63 [SE ± 0.24] active vs 0.33 [SE ± 0.04] remission; *p* < 0.0001, AUC 0.91; mean abdominal/S 1.03 [SE ± 0.24] vs 0.37 [SE ± 0.12]; *p* = 0.009, AUC 0.71). Therapy was escalated in 12 patients (3 CD and 9 UC) due to disease relapse. Therapy escalation resulted in the reduction of PRO/SS as evaluated at the subsequent phone consultation.

**Conclusions:**

PRO/SS might represent a feasible tool to evaluate disease activity and therapy outcome in IBD patients during periods of limited access to outpatient clinics.

**Supplementary Information:**

The online version contains supplementary material available at 10.1186/s41687-021-00323-z.

## Introduction

The COVID-19 pandemic outbreak, declared on March the 11th 2020 by the World Health Organization, has suddenly generated the need to apply distance between persons in order to limit infection. This new, ever experienced need, has had a great impact in virtually all human activities including health care. All non-essential medical activities including scheduled in person visits of patients affected by inflammatory bowel disease (IBD) have been delayed or rescheduled [1]. The tight follow-up of IBD patients by planned clinical evaluations and biomarkers monitoring (e.g. faecal calprotectin, C-reactive protein (CRP)), is considered a standard of care [2]. During the lock down, IBD patients were at risk of suboptimal management since they were admitted to hospital only after overt disease relapse [3, 4]. Moreover, the risk of infection and COVID-19 development by IBD patients under concomitant immunosuppressive and biologic therapies remains unclear [5, 6]. Recent data from a national registry of COVID-19 cases among the IBD population suggests that active disease might be a risk factor for developing COVID-19 (OR 10.25 [95% CI 2.11–49.73]) [1, 7]. Accordingly, the international organization for the study of the inflammatory bowel disease (IO-IBD) gave indication not to interrupt immunosuppressive and biologic therapies for the risk of disease relapse [8].

In order to guarantee clinical assistance to patients affected by IBD, during the lock down, in person visits were converted into phone consultations in many IBD centers as recently reported [9, 10]. However, how to collect clinical data for disease activity assessment in this setting is poorly defined. Crohn’s disease (CD) and ulcerative colitis (UC) Patients-Reported Outcomes Signs and Symptoms (CD- and UC-PRO/SS), are validated measure systems recently developed to assess disease activity from patient’s perspective according with the Food and Drug Administration (FDA) guidelines for the development of PRO measures [11, 12].

At our center, from March to May 2020, nearly all scheduled in-person visits were converted into remote consultations by phone calls and CD- and UC-PRO/SS measures were used to assess disease activity of IBD patients. The aim of this study was to assess the feasibility and validity of CD- and UC-PRO/SS to evaluate disease activity by remote and to guide therapy interventions in case of disease relapse.

## Methods

### Patients

All in-person visits scheduled between March and May 2020 for IBD patients, both CD and UC, in active follow-up at the IBD center of ******************* were converted to phone consultations. Patients were consecutively contacted and those who met inclusion criteria were enrolled in the study. Inclusion criteria included age equal or greater than 18 years with an established diagnosis of either CD or UC for at least 6 months. Unclassified IBD (IBD-U) and patients with ostomy were excluded from the study. Patients with severe disease relapse were also excluded from the study and invited to have an in-person evaluation. All patients were made aware of the restrictions adopted by the hospital to limit non-urgent in-person visits due to COVID-19 pandemic and had to agree to be interviewed by phone about their clinical status. Informed written consent on recording of personal data for scientific purposes was obtained by all patients.

### Disease relapse definition and management

Disease relapse and remission were defined by global interpretation of clinical information retrieved during phone consultation and results from blood and faecal tests when available. Altered faecal calprotectin was defined if ≥250μg/kg [13]. The definition of disease relapse was given in agreement by two IBD specialists involved in the visit. Patient’s therapy was escalated or de-escalated according to the resulting global evaluation. For those patients who needed therapy escalation or de-escalation, therapy modification was discussed with the patient before the indication was given. In case of new indication or modification of concomitant biologic therapy, decision was confirmed after in-person clinical evaluation. In patients who needed therapy escalation at the end of the visit, a new remote consultation was scheduled to evaluate the clinical outcome. During the follow-up visit, PRO/SS and results from blood and faecal tests if available, were reviewed in order to assess the benefit of the therapeutic intervention.

### CD- and UC-PRO/SS

During phone consultations, clinical information were collected according to CD- and UC-PRO/SS (Suppl. Table 1) [11, 12]. CD-PRO/SS considers two modules: bowel signs and symptoms (bowel/SS) composed by three items (i.e. number of bowel movements, mostly liquid bowel movements and need to have bowel movement right away), considering the last week preceding the phone visit, and abdominal symptoms (abdominal/S) composed by three items (i.e. abdominal pain, passing gas and bloating). UC-PRO/SS considers two modules: bowel/SS (i.e. number of bowel movements, number of liquid bowel movements, presence of blood in bowel movements, presence of mucus in bowel movements, leak before reaching toilet and need to have bowel movement right away), considering the last week preceding the phone visit, and abdominal/S composed by three items (i.e. abdominal pain, passing gas and bloating). The average score of all items defines the score of the module. PRO/SS bowel signs and symptoms ranges from 0 to 5.33 and from 0 to 4.67 for CD and UC respectively, while abdominal symptoms ranges from 0 to 4 for both. The score of each item was recorded in the patient’s clinical record as reported by the interviewed patient. Patients had the opportunity to send blood and faecal tests results by email to the dedicated email box of the IBD center. Results from blood and faecal tests were evaluated during the visit. All visits were performed by the same two IBD specialists in tandem.

**Table 1:** Population characteristics. Data are expressed as n (%) or median (IQR)

	CD ***n*** = 21	UC ***n*** = 56
Gender (F)	13 (61.9%)	33 (58.9)
Age	59.1 (45.2–65.8)	56.3 (40.3–64.1)
Disease duration	6 (5–10)	7 (3–12)
Extent		
E1(proctitis and proctosigmoiditis)		8 (14.3%)
E2(left sided colitis)		29 (51.8%)
E3 (pancolitis)		17 (33.9%)
Localization		
L1 (ileal)	13 (61.9%)	
L2 (colic)	3 (14.3%)	
L3 (ileocolic)	5 (23.8%)	
Behavior		
B1 (inflammatory)	12 (57.1%)	
B2 (stenotizing)	6 (28.6%)	
B3 (penetrating)	3 (14.3%)	
Biologics/ISS	9 (42.7%)	4 (7.14%)
Surgery	6 (28.6%)	0 (0%)

### Outcomes

The primary objective of this study was to assess the feasibility of using CD- and UC- PRO/SS to evaluate IBD clinical activity by remote. In order to do this, the following end-points were considered: the rate of satisfying interviews in which data collection was considered complete and accurate, the difference of PRO/SS score between patients defined as having active disease and those judged in remission by global clinical assessment, the difference of PRO/SS before and after therapy intensification in case of disease relapse. Accuracy of PRO/SS in classifying patients with active or inactive disease was also calculated. The study was carried out according to the clinical practice adopted during the re-modulation of clinical activities arrogated during the lock down. No additional procedures were required for the sake of the study alone. The study adhered to the Declarations of Helsinki. The study was approved by the Independent Ethic Committee of the University Hospital of University Hospital of Cagliari (PG/2020/19837).

### Data analysis

Descriptive statistics were used to describe demographic and clinical characteristics of patients. Non parametric continuous variables were calculated using the Mann-Whitney U test and reported as median and interquartile range (IQR), parametric continuous variables were calculated using the paired T test and reported as mean ± SE, categorical variables were expressed as absolute numbers and percentage. The normal distribution was assessed by Kolmogorov-Smirnov test. Differences of PRO/SS score between patients defined as having clinical relapse and those in remission were assessed by Mann-Whitney test, while variation of PRO/SS before and after therapeutic intervention was assessed by Wilcoxon Signed Rank test. Statistical significance was considered for *p* < 0.05. Receiver operating characteristic (ROC) curves were used to describe the accuracy of PRO/SS for correctly classifying patients in remission. All data were analyzed using IBM SPSS Statistics version 20 (IBM, Costa Mesa, CA USA).

## Results

From March the 3rd to May the 18th, 90 IBD patients with scheduled in-person follow up visits were contacted by phone to assess disease activity. Remote clinical evaluation was successful in 77 (84.6%) patients. In 13 patients evaluation was unsuccessful. Eight patients (8.8%) did not answer or refused to be interviewed by phone. Three patients (3.3%) had incomplete interviews due to communication issues. Diagnosis of either CD or UC was still uncertain in two patients and they were excluded from the analysis. Of the 77 patients with satisfying remote evaluation, 21 (27.3%) CD and 56 (72.7%) UC, the median age was 59.1 (IQR 45.2–65.8) for CD patients and 56.2 (IQR 40.3–64.1) for UC patients, Table 1. The median disease duration was 6 (IQR 5.0–10.0) and 7 (IQR 3.0–12.0) years for CD and UC respectively. Thirteen CD patients (61.9%) had ileal (L1), 3 (14.3%) colonic (L2) and 5 (23.8%) ileo-colonic (L3) disease localization. Disease behavior was inflammatory (B1) in 12 (57.1%), fibrostenosing (B2) in 6 (28.6%) and penetrating (B3) in 3 (14.3%) patients. Nine CD patients (42.8%) were under biologic and/or ISS treatment. Six CD patients (28.6%) had resective surgery for luminal disease. Forty-six UC patients (85,7%) had left-sided or extended colitis (E2-E3 according to the Montreal classification) and 4 (7.14%) were treated with biologics and/or immunosuppressants (ISS).

### CD-PRO/SS and therapeutic intervention in CD patients

**Fig. 1:**
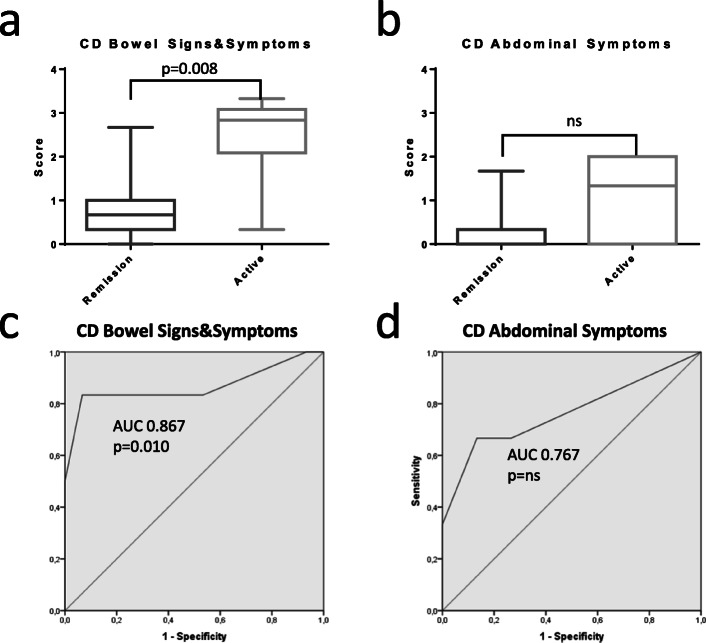
CD bowel signs and symptoms (**a**) and abdominal symptoms (**b**) measures of patients defined in clinical remission or suffering from disease relapse (active) by global clinical assessment. ROC curves of CD bowel signs and symptoms (**c**) and abdominal symptoms (**d**) referred to disease activity status by global clinical assessment. Statistical significance of differences (*p* value) is reported in the plot area. AUC: area under the curve. (ns) not significant

CD-PRO/SS were assessed in all 21 patients included in the analysis. Blood and/or faecal tests were available in 9 (42.8%) patients. Six of 21 (28.6%) patients were considered having active disease. The mean CD-PRO/SS bowel signs/symptoms module in patients with active disease was higher than the one calculated in patients considered in remission (2.50 [SE ± 0.44] active vs 0.76 [SE ± 0.18] remission; Δ = 1,74; *p* = 0.008; Fig. 1A) and the AUC was 0.87 (CI 0.64–1, *p* = 0.01; Fig. 1C). The mean CD-PRO/SS abdominal symptoms was also numerically higher in patients with active disease as compared to patients in remission (1.11 [SE ± 0.38] active vs 0.24 [SE ± 0.13] remission, Δ = 0,87) but the difference did not reach the statistical significance (*p* = 0.066); Fig. 1C-D.

### UC-PRO/SS and therapeutic intervention in UC patients

**Fig. 2:**
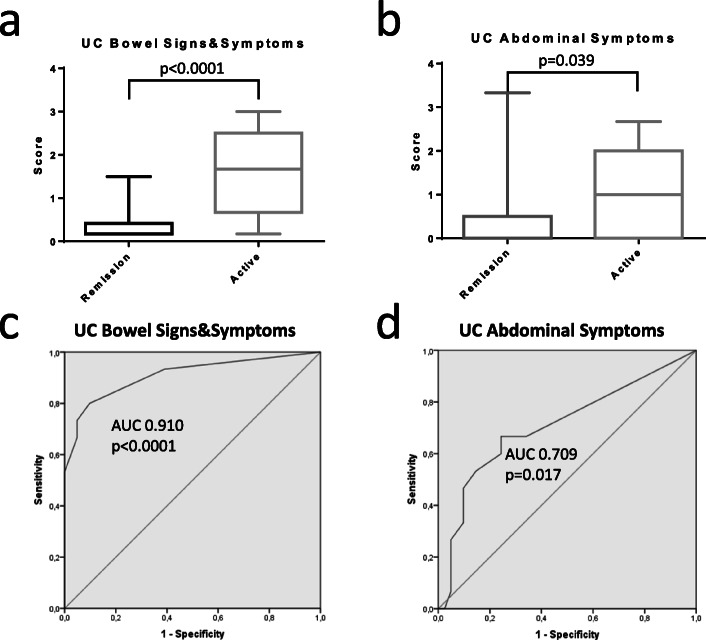
UC bowel signs and symptoms (**a**) and abdominal symptoms (**b**) measures of patients defined in clinical remission or suffering from disease relapse (active) by global clinical assessment. ROC curves of UC bowel signs and symptoms (**c**) and abdominal symptoms (**d**) referred to disease activity status by global clinical assessment. Statistical significance of differences (p value) is reported in the plot area. AUC: area under the curve. (ns) not significant

UC-PRO/SS was assessed in all 56 UC patients included in the analysis. Blood and/or faecal tests were available in 22 (39.3%) patients. Based on the general assessment of patients and available tests, active disease was identified in 15 of 56 (26.8%) patients. The mean UC-PRO/SS bowel signs/symptoms module of patients with active disease was significantly higher than those judged being in clinical remission (1.63 [SE ± 0.24] active vs 0.33 [SE ± 0.04] remission; Δ = 1,3; *p* < 0.0001, Fig. 2A). The AUC of bowel signs and symptoms was 0.91 (CI 0.81–1, *p* < 0.0001; Fig. 2C). The mean UC-PRO/SS abdominal symptoms were also significantly higher in active as compared to inactive disease (1.03 [SE ± 0.24] active vs 0.37 [SE ± 0.12] inactive; Δ = 0,66; *p* = 0.009; Fig. 2B) and abdominal symptoms AUC was 0.71 (CI 0.54–0.87, *p* = 0.017; Fig. 2D).

### Outcome of remote therapy modification

Indication to therapy escalation was given in all CD active patients. Two CD patients (33.3%) received indication to start a biologic therapy and Adalimumab was optimized to every week maintenance regimen in one case. A systemic steroid course was prescribed in another case. One patient affected by fibrostenosing disease refractory to medical therapy was admitted to hospital with sub-occlusive symptoms. One patient refused therapy escalation. No therapy de-escalation was indicated in the group of CD patients. Indication to therapy escalation was given to all UC patients with active disease. Two UC patients (13.3%) received indication to start anti-tumor necrosis factor (TNF) therapy with Infliximab and the indication was confirmed during the following in-person visit. A steroid course was initiated in 9 UC patients (60.0%) while 3 (20.0%) underwent 5-aminosalicydic acid (5-ASA) therapy intensification. One patient refused therapy escalation. Therapy de-escalation consisting in reduction of 5-ASA therapy (i.e. reduction of 5-ASA from 4.8 g to 2.4 g or suspension of local therapy while maintaining oral 5-ASA) was indicated in 3 patients.

**Fig. 3:**
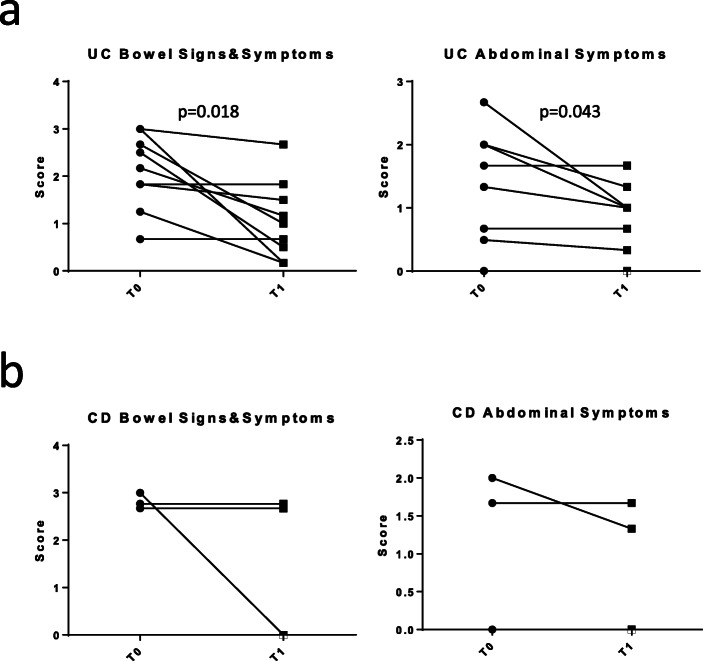
UC- (**a**) and CD- (**b**) bowel signs and symptoms and abdominal signs evaluated before (T0) and after (T1) therapy escalation. Statistical significance (p value) of differences between the two time points is indicated in the plots. (ns) not significant

In order to assess the outcome of therapy intensification, 12 of 21 patients (51,7%; 3 CD and 9 UC) in which therapy was intensified, had a second remote evaluation. The second evaluation was performed after a median time of 30 days (IQR 13–30). Two of three CD patients with therapy escalation at T0 showed clinical improvement at T1. One patient remained stable. The mean CD-PRO/SS bowel signs and symptoms decreased from 2.78 (T0) to 1.78 (T1) while UC-PRO/SS abdominal symptoms decreased from 1.22 (T0) to 1 (T1); Fig. 3B. The insufficient number of intensified CD patients limited further statistical analysis. The second remote evaluation was not available in 9 patients. Six patients were evaluated in-person at the end of the lock down showing disease improvement, 2 patients initiated a biologic therapy after the end of the lock down and one patient who refused therapy escalation was lost at follow up. Seven of 9 UC patients (77.7%) in which therapy was intensified at T0 showed clinical improvement at the second evaluation performed by remote (T1). Two patients remained stable. Accordingly, the mean UC-PRO/SS bowel signs and symptoms in patients who had therapy intensification decreased from 2.10 at T0 to 1.07 at T1 (− 1.03, 95%CI − 1.78 to − 0.27; *p* = 0.018), while the mean PRO/SS abdominal symptoms decreased from 1.20 at T0 to 0.78 T1 (− 0.43; − 0.88 to − 0.02; *p* = 0.043; Fig. 3A). Patients considered to be in clinical remission did not receive a second phone call. However, they were invited to contact our center in case of symptoms recurrence. Of note, no patient among those considered to be in clinical remission contacted our center or had the necessity to go to the emergency department for any disease-related cause after the first phone call.

## Discussion

At the time this manuscript is written, new lock downs have been adopted in many countries around the world due to the COVID-19 pandemic, renewing the question about how to measure disease activity and manage IBD patients by remote. Studies exploring the feasibility of telemedicine in IBD are few and none of them dealt with the sanitary condition we are facing at the moment. In a Danish cohort of mildly-to-moderately active UC patients aiming at evaluate the impact of disease self-monitoring on patient’s adherence to therapy [14], disease activity was measured using self-administered Simple Clinical Colitis Activity Index (SCCAI) [15]. The same group demonstrated a significant reduction of SCCAI in the web-based management group of UC patients treated with MMX-mesalamine as compared to the standard group [16]. SCCAI considers exclusively clinical items but a sub-optimal correlation between the online patient self-administered- and the in-person IBD specialist evaluated- SCCAI was shown [17]. In another study, the Harvey Bradshow Index (HBI) was used to assess disease activity of CD patients by remote [18]. Similarly to SCCAI, HBI has not been developed for self-evaluation of IBD activity. In a multicenter trial, IBD patients were randomized to mobile application-based monitoring (TELE-IBD weekly or EOW) or in-person visits for 12 months [19]. Clinical activity was measured using self-administered SCCAI and HBI. In a study involving IBD patients randomized to web-based monitoring once every three months with frequency intensification in case of disease flare or to standard in-person care for 12 months [20], disease activity was assessed by the Monitor IBD At Home (MIAH) questionnaire, a patient-reported symptom-based measure validated to endoscopy [21].

Collectively, these studies explored the potential of telemedicine and telehealth to improve disease control, increase patient’s empowerment and reduction of health-care utilization in normal times. Though, the effectiveness of such approaches cannot be directly translated in the situation we are facing during the COVID-19 pandemic for several reasons. First of all, the main aim of the aforementioned studies was to assess whether telemedicine could reduce the number of in-person visits in order to reduce the consumption of health care resources. In this moment, we need to know whether remote monitoring can substitute in-person visits to guide therapeutic interventions. Secondly, in most of the studies patients were affected by mildly-to-moderately active disease and these patients might not represent the totality of patients that we might need to switch to remote consultation during the lock down. Finally, none of these studies focused on how to measure disease activity by remote. Indeed, in most of the telemedicine studies, clinical activity was assessed by self-administered clinical scores designed for in-person evaluation.

In our study we prospectively evaluated patients by phone consultations using the CD- and UC-PRO/SS, patient reported outcome measures [11, 12]. The use of the PRO/SS bowel signs and symptoms and the abdominal symptoms modules allowed us to standardize the way signs and symptoms were collected using words and terms easy to understand by patients. In our cohort, the use of PRO/SS was possible in more than 80% of patients indicating a good acceptance rate among patients. Unsuccessful cases were related to communication issues mostly related to the age of the patients and education level. The bowel signs and symptoms module showed a good performance in identifying disease activity as defined by clinical global assessment as indicated by AUC (CD bowel/SS 0.86; UC bowel/SS 0.91). In contrast, the performance of the abdominal symptoms module was lower. This might be due to the absence of objective items in the abdominal symptom module as compared to the bowel signs and symptoms. These data suggest that the module bowel signs and symptoms rather than abdominal symptoms should be included in the phone evaluation.

Indication to therapy escalation was given in one third of patients. Half of them had a second phone evaluation after therapy escalation showing improvement of clinical status by PRO/SS; patients considered to be in clinical remission did not receive a second phone call. However, they were invited to contact our center in case of symptoms recurrence. Of note, no patient among those considered to be in clinical remission contacted our center or had the necessity to go to the emergency department for any disease-related cause after the first phone call. This further indirectly confirm the validity of PROs as a valuable screening tool in this subset of patients. Moreover, despite the small number of patients with therapy escalation does not allow to draw conclusions, these data suggest that PROs used during the phone evaluation might be useful to monitor clinical intervention in relapsing IBD patients.

At the best of our knowledge this is the first study analyzing the use of validated PROs to evaluate clinical activity of IBD patients by remote during the SARS-CoV2 pandemic. However, our study presents some relevant limitations. First of all, the small sample size. The number of patients enrolled in the study was determined by the number of visits scheduled during the lockdown period and was not calculated on the basis of predefined outcomes. Nevertheless, the objective of the study was to assess the feasibility of using PRO/SS during remote evaluation of IBD patients in an emergency setting, and as a pilot study a predetermined sample size was not calculated. Despite the small sample size, CD location and behavior rates resulted similar to those reported in the literature according to the disease duration [22]. In contrast, UC proctitis and proctosigmoiditis were slightly under- and over-represented respectively [23]. This might be due to the fact that patients with more extensive and severe disease are in active follow-up at our regional referral center.

Patients affected by severely active disease were not evaluated by telemedicine and were excluded because considered urgent conditions that would have theoretically needed hospitalization. Indeed, they were the only patients authorized by local authorities to perform in-site visits and to have hospital access for granted. PRO/SS has not been validated for endoscopic activity and faecal calprotectin or blood tests were not available for all patients due to the limited access to clinical laboratory during the lock down. Therefore, an objective evaluation of disease activity supporting clinical decisions was not always available. Finally, the number of patients requiring therapy escalation was limited, thus preventing the possibility to draw clear conclusions on the capacity of PRO/SS to guide therapeutic intervention.

In conclusion, this study was intended to be a pilot experience in an emergency era that, at least at the moment we are writing, is not expected to resolve soon. Accordingly, in the perspective of a still ongoing and future need of remote patient care, here we report a real-life experience of remote follow-up of IBD and provide evidence that the use of CD- and UC-PRO/SS to assess IBD patient’s disease activity is feasible/usable and might monitor therapy intervention in case of disease flare.

## Supplementary Information

Additional file 1

## Data Availability

The datasets used and/or analysed during the current study are available from the corresponding author on reasonable request.

## Abbreviations

IBD: inflammatory bowel disease
PRO: patient-reported outcome
CD: Crohn’s disease
UC: ulcerative colitis
ISS: immunosuppressant
HBI: Harvey-Bradshow Index
SCCAI: simple clinical colitis activity index
