# Supplementary material for: Crohn’s disease and ulcerative colitis patient-reported outcomes signs and symptoms for the remote management of inflammatory bowel disease during the COVID-19 pandemic
Source: J Patient Rep Outcomes. 2021 Jun 24;5:48. doi: 10.1186/s41687-021-00323-z (PMC8223182; doi:10.1186/s41687-021-00323-z)
Supplement: Supplementary file 1 — Additional file 1 [file 41687_2021_323_MOESM1_ESM.docx]

Suppl. Table 1

**UC-PRO/SS – Bowel Signs and symptoms**

| Item | Range |
| --- | --- |
|  |  |
| Number of bowel movements* | 0 – 8 |
| Number of liquid bowel movements** | 0 – 4 |
| Blood in bowel movements** | 0 – 4 |
| Mucus in bowel movements** | 0 – 4 |
| Leak before reaching toilet** | 0 – 4 |
| Need to have bowel movement right away | 0 – 4 |
| *1 (0), 2 (1-2), 3 (3-4), 4 (5-6), 5 (7-9), 6 (10-12), 7 (13-17), 8 (18-24) considering the last week preceding the phone visit  ** 0 (never or none), 1 (rarely or mild), 2 (sometimes or moderate), 3 (often or severe), 4 (always or very severe) considering the last week preceding the phone visit | |

**UC-PRO/SS – Abdominal Symptoms**

| Item | Range |
| --- | --- |
|  |  |
| Passing gas* | 0 – 4 |
| Pain in belly* | 0 – 4 |
| Bloating in belly* | 0 – 4 |
| * 0 (never or none), 1 (rarely or mild), 2 (sometimes or moderate), 3 (often or severe), 4 (always or very severe) considering the last week preceding the phone visit | |

**CD-PRO/SS – Bowel Signs and symptoms**

| Item | Range |
| --- | --- |
|  |  |
| Number of bowel movements* | 0 – 8 |
| Mostly liquid bowel movements** | 0 – 4 |
| Need to have bowel movement right away** | 0 – 4 |
| *1 (0), 2 (1-2), 3 (3-4), 4 (5-6), 5 (7-9), 6 (10-12), 7 (13-17), 8 (18-24) considering the last week preceding the phone visit  ** 0 (never or none), 1 (rarely or mild), 2 (sometimes or moderate), 3 (often or severe), 4 (always or very severe) considering the last week preceding the phone visit | |

**CD-PRO/SS – Abdominal Symptoms**

| Item | Range |
| --- | --- |
|  |  |
| Passing gas* | 0 – 4 |
| Pain in belly* | 0 – 4 |
| Bloating in belly* | 0 – 4 |
| * 0 (never or none), 1 (rarely or mild), 2 (sometimes or moderate), 3 (often or severe), 4 (always or very severe) considering the last week preceding the phone visit | |
